# Authors' Reply: Response to Ian Clark

**DOI:** 10.1371/journal.pmed.0030069

**Published:** 2006-01-31

**Authors:** Arjen Dondorp, Nick White, Nick Day

**Affiliations:** **1**Mahidol UniversityBangkokThailand

## Abstract

n/a

We are grateful for Ian Clark's suggestions [ 1] in response to our research article [ 2]. Much remains to be learned about the pathogenesis of cerebral malaria. It is certainly hard to disprove that triggering local immune responses contributes in some way to the pathophysiology of cerebral malaria. Local overproduction of NO, HMGB1, cytokines, or other mediators yet to be discovered could impair neurotransmission. But their roles remain hypothetical, and thus far, none of the proposed hypotheses have passed the stage of showing a correlation between the severity of disease and the proposed mediator. A role in murine malaria pathogenesis cannot be translated directly to human pathophysiology since the basic pathophysiological phenomena are essentially different in these animals. Although the concept of impairment of microcirculation in severe malaria by sequestered parasitized erythrocytes causing local tissue dysoxia, acidosis, and metabolic dysfunction is a simple one, there is considerable evidence that it is correct. Lactate/pyruvate ratios are, in contrast with sepsis, clearly increased in severe malaria, which is compatible with anaerobic glycolysis as a source of lactate production [ 3]. Of the human malarias, only Plasmodium falciparum sequesters in vital organs, and this is also the species responsible for the vast majority of malaria-related deaths. Autopsy studies of fatal malaria cases show convincing correlations between extent of sequestration in the brain and coma as a presenting symptom [ 4]. Direct visualisation of the microcirculation in patients with severe malaria shows blockage of capillaries, which become patent after the patient's recovery. Strong support for the central role of parasite sequestration in the pathophysiology of lethal malaria comes from the largest trial ever conducted on severe malaria, which showed that artesunate reduces mortality by 34% compared with quinine [ 5]. Both artesunate and quinine are very active against sequestered parasites, preventing their development to schizonts, but unlike quinine, artesunate is also active against the younger forms of the parasite, preventing their maturation and sequestration in the microcirculation of vital organs. The greatest mortality benefit in this trial compared with quinine was in patients with high parasitaemias, indicating that prevention of sequestration (rather than prevention of schizont rupture) saved lives.
